# Leflunomide is equally efficacious and safe compared to low dose rituximab in refractory rheumatoid arthritis given in combination with methotrexate: results from a randomized double blind controlled clinical trial

**DOI:** 10.1186/s12891-017-1673-3

**Published:** 2017-07-19

**Authors:** Harindu Wijesinghe, Priyadharshini Galappatthy, Rajiva de Silva, Suranjith L. Seneviratne, Ushagowry Saravanamuttu, Preethi Udagama, Melanie Hart, Peter Kelleher, Upul Senerath, Rohini Fernandopulle, Lilani P. Weerasekera, Lalith S. Wijayaratne

**Affiliations:** 1 Department of Rheumatology, The National Hospital of Sri Lanka, Colombo, Sri Lanka; 2 Department of Pharmacology, Faculty of Medicine, University of Colombo, Colombo, Sri Lanka; 3 Department of immunology, Medical Research Institute, Colombo, Sri Lanka; 4 Consultant and Professor in Clinical Immunology and Allergy, Royal Free Hospital, University College London, London, UK; 5 Department of Surgery, Faculty of Medicine, University of Colombo, Colombo, Sri Lanka; 6 Department of Zoology, Faculty of Science, University of Colombo, Colombo, Sri Lanka; 7 Immunology Section Imperial College, and Department of Infection & Immunity, London, UK; 8 Department of Community Medicine, Faculty of Medicine, University of Colombo, Colombo, Sri Lanka; 9 Department of Pharmacology, General Sir John Kotelawala Defense University, Colombo, Sri Lanka; 10 Department of Pharmacology and Pharmacy, Faculty of Medicine, University of Colombo, PO Box 271, Kynsey Road, Colombo 8, Sri Lanka

**Keywords:** Rheumatoid arthritis, Rituximab, Leflunomide, Clinical trial, Biologic Disease Modifying Anti Rheumatic Drugs (DMARDS)

## Abstract

**Background:**

The standard dose of rituximab used in rheumatoid arthritis (RA) is 1000 mg but recent studies have shown that low dose (500 mg) is also effective. Efficacy of low dose rituximab in rheumatoid arthritis (RA) refractory to first-line non-biologic Disease Modifying Anti Rheumatic Drugs (DMARDs), compared to leflunomide is unknown. In a tertiary care referral setting, we conducted a randomized, double blind controlled clinical trial comparing the efficacy and safety of low-dose rituximab-methotrexate combination with leflunomide-methotrexate combination.

**Methods:**

Patients on methotrexate (10-20 mg/week) with a Disease Activity Score (DAS) > 3.2 were randomly assigned to rituximab (500 mg on days 1 and 15) or leflunomide (10-20 mg/day). The primary end-point was ACR20 at 24 weeks. Sample of 40 had 70% power to detect a 30% difference. ACR50, ACR70, DAS, EULAR good response, CD3 + (T cell), CD19 + (B cell) and CD19 + CD27+ (memory B cell) counts, tetanus and pneumococcal antibody levels were secondary end points.

**Results:**

Baseline characteristics were comparable in the two groups. At week 24, ACR20 was 85% vs 84% (*p* = 0.93), ACR50 was 60% vs. 64% (*p* = 0.79) and ACR70 was 35% vs 32% (*P* = 0.84), in rituximab and in leflunomide groups respectively. Serious adverse events were similar.

With rituximab there was significant reduction in B cells (*p* < 0.001), memory B cells (*p* < 0.001) and pneumococcal antibody levels (*P* < 0.05) without significant changes in T cells (*p* = 0.835) and tetanus antibody levels (*p* = 0.424) at 24 weeks. With leflunomide, significant reduction in memory B cells (*p* < 0.01) and pneumococcal antibody levels (*p* < 0.01) occurred without significant changes in B cells (*P* > 0.05), T cells (*P* > 0.05) or tetanus antibody levels (*P* > 0.05).

**Conclusions:**

Leflunomide-methotrexate combination is as efficacious as low-dose rituximab-methotrexate combination at 24 weeks, in RA patient’s refractory to initial DMARDs. The high responses seen in both groups have favorable cost implications for patients in developing countries. Changes in immune parameters with leflunomide are novel and need further characterization.

**Trial registration:**

The trial was registered with the Sri Lanka Clinical Trials Registry (SLCTR), a publicly accessible primary registry linked to the registry network of the International Clinical Trials Registry Platform of the WHO (WHO-ICTRP) (registration number: SLCTR/2008/008 dated 16th May 2008).

## Background

Rheumatoid arthritis (RA) is a crippling disease associated with significant morbidity and mortality. Disease-Modifying Anti-Rheumatic Drugs (DMARDs), are used to control the disease as well as to slow the disease progression. Despite the use of these agents, some patients continue to have persistently active disease [1].

The introduction of biologic DMARDs has helped those not responding to non-biologic DMARDs. Biologic DMARDs recommended by current guidelines and are in use include, Tumor Necrosis Factor inhibitors (TNFi), and non TNF alpha agents, rituximab (anti CD-20 monoclonal antibodies), abatacept (inhibitor of Cytotoxic T lymphocyte Antigen-4 (CTLA4) co-stimulation) and tociluzimab (IL-6 inhibitor) [2].

Current treatment guidelines recommend monotherapy, usually with methotrexate as the first line DMARD, but recommend combinations of DMARDs in those with high disease activity, in the presence poor prognostic factors and in non-responding patients. Due to fears of hepatotoxicity combination therapy with methotrexate and leflunomide has not been used frequently [3]. Recommended next line of treatment in DMARD combination failures are the TNFi. Some data also suggest that adding a TNFi early in active disease is superior to adding conventional DMARDs to methotrexate therapy [4].

TNFi are the first line biologic agents recommended as they give long term efficacy and safety data from clinical trials and clinical registries [5]. However risk of reactivation of tuberculosis (TB) especially in populations from TB endemic regions such as South Asia remains a concern when using a TNFi in this region [6–9]. Moreover TNFi in recommended dosages are expensive and are not available routinely through the government health services.

The use of rituximab as a first line biologic agent has recently gained acceptance [10] and it is now a licensed indication for rheumatoid arthritis [10, 11]. Rituximab is effective in both methotrexate non-responders [12] as well TNFi non-responders [13] and has the advantage of needing only two infusions for a long lasting remission. Although initially recommended for use in TNFi non-responders, current evidence from clinical trials, and changes in recommendations [2] have promoted it as second line therapy after failure of non-biologic DMARDs. Moreover, not reactivating latent TB makes it a suitable biologic to use in TB endemic regions of the world [14].

The usually recommended dose of Rituximab is 1000 mg but some clinical trials have shown that the dose of 500 mg to be efficacious as well [15, 16]. Data from the MIRROR study [15] and SERENE study [17] suggested that overall efficacy of two infusions of rituximab 500 mg and two infusions of 1000 mg could not be clearly differentiated, although some of the efficacy end points suggested improved outcomes in the rituximab 1000 mg group [15]. More recently, a systematic review of 6 clinical trials and 2 cohort studies [18] have shown similar efficacy of low dose rituximab compared to high dose regimen. Another study on international cohort data of a large number of patients who have received rituximab has also given similar findings [19].

Although available trial data favors leflunomide-methotrexate combinations in non-responders to conventional DMARD combinations [20, 21], only a few clinical trials have addressed adequately the efficacy and safety of this cheaper alternative. The recently published SMILE study has given evidence on the safety of combining lefluonomide with methotrexate as this combination was well tolerated with adverse effect profile comparable to monotherapy with either methotrexate or leflunomide [22]. Efficacy data from South Asian patients using the cheaper low dose rituximab-methotrexate regimen and the even more cheaper leflunomide-methotrexate combination is not available. To our knowledge no previous trial has compared the leflunomide-methotrexate combination versus a biologic agent given with methotrexate in refractory RA.

To address these questions we carried out a randomized double blind controlled clinical trial to study the efficacy and safety of low dose rituximab-methotrexate combination compared to leflunomide-methotrexate combination in patients with rheumatoid arthritis not responding to initial treatment with non-biologic DMARDs.

## Methods

### Patients

Patients were recruited from the two-rheumatology clinics at the National Hospital of Sri Lanka (NHSL). The patients were included if they were older than 18 years, fulfilled the revised 1987 ACR criteria for diagnosis of rheumatoid arthritis [23], and had active disease despite treatment with non biologic DMARDs containing at least 10 mg of methotrexate per week for more than 6 months. Active disease was defined by the presence of at least four swollen and four tender joints and a raised serum C-reactive protein (CRP) level of ≥0.6 mg/dl, and erythrocyte sedimentation rate (ESR) of >28 mm per hour. Patients were on stable doses of non-steroidal anti-inflammatory drugs (NSAIDS) or corticosteroids not exceeding 10 mg per day of prednisolone (or the equivalent) for at least 3 months. Patients previously given biologic DMARDS or leflunomide were excluded from the study.

Patients were also excluded if they had an autoimmune disease other than rheumatoid arthritis, ACR functional class IV disease, an active infection and history of recurrent clinically significant infections or recurrent bacterial infections. Screening for tuberculosis was not done, as rituximab has not been shown to reactivate tuberculosis.

### Study protocol

The ethics committee of the Faculty of Medicine, University of Colombo, Sri Lanka, approved the study. The trial was registered prior to commencement with the Sri Lanka Clinical Trials Registry (SLCTR), a publicly accessible primary registry (www.slctr.lk) recognized by the World Health Organization, and the protocol can be accessed through this registry. Written informed consent was obtained from all study participants.

At study entry, all patients were taking methotrexate at a stable dose of 10-20 mg for at least 12 weeks. Forty patients were randomly assigned to the two groups using block randomization. One group received two infusions of rituximab 500 mg reconstituted to 500 ml 0.9% sodium chloride intravenous infusions on days 0 and days 14, together with placebo tablets manufactured matching to yellow colored leflunomide tablets. The second group received leflunomide10mg as starting dose increased to 20 mg, in the absence of elevation of liver enzymes with a placebo infusion of 500 ml of 0.9% sodium chloride. Loading dose of leflunomide was not given since it has not shown any advantage over standard maintenance dose [24]. Methotrexate, steroids and NSAID’s were continued in stable doses in both groups. Any other DMARDs that the patient was using (eg. sulphasalazine, hydroxychloroquine) were stopped prior to randomization. Both groups received a 14-day course of steroids, 100 mg of methylprednisolone 2 h prior to the infusion and prednisolone 60 mg daily (on days 2 to 7) and 30 mg daily (on days 8 to 14), similar to steroids regimens used in the initial rituximab trials [12]. Patients were subsequently continued on their maintenance steroid doses. Premedication with IV chlorpheniramine 4 mg and oral paracetamol 1 g were given prior to each infusion to both groups.

Patients were screened and enrolled into the study based on inclusion and exclusion criteria and were given a study number in the order that they entered the study by HW. Random allocation sequence was generated by PG using excel random sequence generator version 1.0 using blocks of 10 (block randomisation) to allocate patients in 1:1 ratio to the two groups ensuring equal numbers are allocated to the two groups. Forty opaque sealed envelopes were prepared, indicating the allocation of each randomised patient. The envelopes and the allocation sequence were kept under lock and key with one investigator (PG) who gave envelopes to study coordinators when each patient was enrolled into the study, for preparation of rituximab or placebo infusion. Leflunomide, which came as foil packed identifiable tablets were removed from foil and were put into air tight containers containing one months supply, with label of the bottle containing the patient ID. Identical looking yellow coloured placebo tablets manufactured by the same company manufacturing leflunomide were put into airtight containers similarly labelled with one month supply, which were given to patients randomised to rituximab. Study nurses and pharmacists prepared, supplied and administered the study drugs to the patients, supervised by unblinded study coordinators ensuring accurate study drug administration. Patient assessment was done by investigators (LSW and LW) who were blinded to treatment. Blinding of patients was maintained throughout the study with administration of matching placebo tablets and 0.9% saline to leflunomide and rituximab arms respectively as described.

### Outcome assessments

Patients were assessed at weeks 4, 8, 12, 16, 20, and 24. Patients and those assessing clinical response (LSW and LW) remained blinded to the treatment. At each visit, history, physical examination and the ACR core set of disease-activity measures and DAS 28 scores were assessed. This included swollen joints count (66 joints), tender joint count (68 joints), patient’s assessment of pain on a scale from 0 (no pain) to100 (unbearable pain), patient’s global assessment of disease activity on a scale from 0 (disease inactive) to 100 (maximal disease activity), physician’s assessment of disease activity and patient’s assessment of physical function by health-assessment questionnaire (HAQ). Disease-activity score, DAS 28 (with physician’s assessment of 28 joints and patient’s self-assessment of disease activity), and the response according to the criteria of the European League Against Rheumatism (EULAR response) were also measured.

Laboratory monitoring included measurement of inflammatory markers, blood counts and routine biochemistry. Baseline and 24 week B cell counts, memory B cell (CD19 + 27+) percentages and T cell counts were measured using a Coulter Epics XL flow cytometer. Pneumococcal and tetanus antibody titers were measured at baseline and at 24 weeks by Enzyme linked immunosorbent assay (ELISA). Patients were followed for up to 48 weeks for adverse effects.

### End points

The primary endpoint was 20% improvement in the American College of Rheumatology core set of disease-activity measures (ACR 20 response) response at 24 weeks. The secondary outcomes included changes in 50% and 70% improvement (ACR50 and ACR70 scores respectively), DAS, EULAR disease score and changes in different immune markers.

### Statistical tests and analysis

Sample size and power of the study was calculated based on the proportion of patients achieving ACR 20 in either group. Assuming that the proportion of patients receiving methotrexate-leflunamide group achieving an ACR 20 response at week 24 would be 25% and the proportion of patients reaching ACR 20 in methotrexate-rituximab group would be 55% (observed with 500 mg rituximab in study by Emery et al.) [16], total of 38 patients was required to detect a 30% difference between the two groups, giving 70% power for a significance level of 0.05. Therefore 40 patients with DAS of more than 3.2 were randomly assigned to receive leflunomide (10-20 mg/day) or rituximab (500 mg on days 1 and 15) added to methotrexate(10-20 mg/week).

The primary analyses were based on intention- to-treat analysis. Statistical analyses were done using non-parametric tests, Mann-Whitney U and Wilcoxan Rank Sum test for paired comparisons and Chi square test for comparison of categorical variables using SPSS 15. An investigator with expertise in statistics did the statistical analyses (US). The local agents of rituximab provided the study drugs, some laboratory reagents and a study coordinator, and no payment was made to the patients or the investigators. The investigators independently developed the trial protocol, collected data, analysed the results and wrote the manuscript.

## Results

**Fig. 1:**
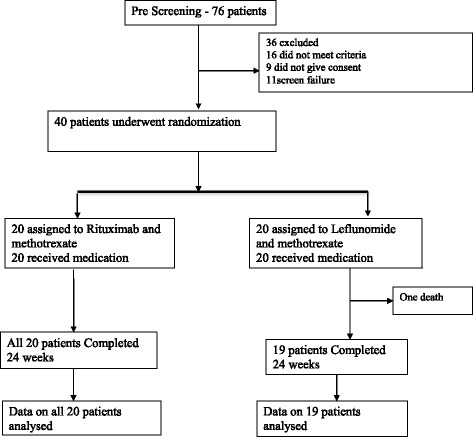
Consort flow diagram of participants at each stage

Flow diagram of participants at each stage of the trial is given in Fig. 1. Baseline characteristic were comparable among the 40 patients in the two groups with no significant differences, except for the mean disease duration, which was longer in the leflunomide group [Table 1]. The study population was primarily female and had RA for a mean duration of 67 months. All patients had similar disease activity with a DAS score of >5.1 at enrolment.

**Table 1:** Baseline Characteristics of the Patients in the two groups

	Rituximab Group (*n* = 20)	Leflunomide Group (*n* = 19)	*P* value
Mean Age in Years(+/−SD)	44.15(±12)	48.2(±10)	0.318
Female sex(%)	16(80%)	18(95%)	0.169^a^
Mean Disease duration in months(+/−SD)	67.9 (±80)	102.8 (±63)	0.034
Mean number of swollen joints (+/−SD)	9.35(±4)	7.58(±4)	0.074
Disease activity Score (DAS) (+/−SD)	6.88(±1)	6.43(±0.5)	0.067
Mean methotrexate dose in mg	17	16	0.67
Prednisolone dose	8.0 mg	7.76 mg	0.730
Patients who were on sulphasalazine and or hydroxychroloquine	20	19	NT
Patients with erosions on plain X Ray	4 (20%)	5 (26%)	0.640
HAQ score	8.23 (±3.36)	8.07 (± 3.37)	0.843
Tender joint count	16.35(± 6.31)	12.89(5.62)	0.955
Pain score (out of 100)	68.50 (±22.64)	72.53 (±15.70)	0.723
Patient assessment score (out of 100)	67.4 (±19.02)	64.37 (± 15.18)	0.743
Physician assessment score (out of 100)	68.85(±16.96)	64.05 (± 15.09)	0.3539
ESR*	71.90 (±36.41)	62.05 (±28.92)	0.473
CRP**	30 (0-600)	18 (0-140)	0.20
RF***	256 (0-1024)	128 (0-512)	0.524

*n* number of patients, *SD* Standard Deviation, *RF* Rheumatoid Factor, *NA* Not applicable. *P* values for difference between means were compared using Mann Whitney U test ^a^For comparison of gender, Chi-square test was used for categorical variable. *ESR was measured in mm/h. **Assay cut off for CRP = 6 mg/L and tested using particle agglutination test, ***Assay cut off for RF = 20 IU/ml and tested using particle agglutination test. NT- Not tested (significance cannot be tested since one value is 100%)

The primary endpoint ACR20, at 24 weeks in the rituximab and leflunomide groups were 85% and 84% respectively, and this difference was not statistically significant [Table 2].

**Table 2:** Clinical Responses at Weeks 24 and mean DAS at baseline

ACR Response	Rituximab Group (*n* = 20)	Leflunomide Group(*n* = 19)	*P* value
No ACR response	3 (15%)	3 (16%)	0.93
ACR 20	17 (85%)	16(84%)	0.93
ACR 50	12 (60%)	12 (64%)	0.79
ACR 70	7 (35%)	6(32%)	0.84
Mean DAS at base line	6.88 (±0.93)^a^	6.43(±0.46)^b^	.067*
Mean DAS at 24 weeks	3.26 (±0.74)^a^	3.25 (±1.02)^b^	0.84*
DAS remission <2.6	4 (20%)	5 (26%)	0.640
DAS low activity <3.2	8 (40%)	8 (42%)	0.894
DAS Moderate activity(3.2-5.1)	12 (60%)	11 (58%)	0.894
DAS High activity (>5.1)	0 (0%)	0 (0%)	NT
EULAR response moderate	12 (60%)	11(58%)	0.894
EULAR good response	8 (40%)	8 (42%)	0.894
HAQ score	2.872 (±2.087)	2.132 (±1.240)	0.388
Tender joint count	1.80 (± 2.26)	1.16 (1.74)	0.564
Pain score	23.70 (±22.64)	22.7 (±18.20)	0.499
Physician global assessment score	11.75 (±10.51)	15.74 (±17.62)	0.983
Patient global assessment score	20.25(±16.12)	20.0 (±15.81)	0.927
Anti-Pneumococcal antibody (units/ml)	132.05 (±81.3)	116.7 (±82.1)	0.429
Anti tetanus antibody	0.760 (±0.43)	0.842(±0.32)	0.7414^#^
CD3	1624.4 (±847.1)	1811.1 (±837.5)	0.50^#^
CD19	42.38 (±62.6)	209 (±164.6)	0.0002^#^
CD27	10.1 (±8.7)	49.38 (±37.9)	0.0061^#^
ESR*	28.05(±16.55)	30.42(±18.42)	0.535
CRP**	6 (0-84)	3(0-54)	0.7
RF***	84(0- 372)	60 (0-720)	0.92

*ACR* American College of Rheumatology, *DAS* Disease activity Scores, *EULAR* European League Against Rheumatism good response criteria**,** *NT* Not Tested. *ESR was measured in mm/h. **Assay cut off for CRP = 6 mg/L and tested using particle agglutination test, ***Assay cut off for RF = 20 IU/ml and tested using particle agglutination test. Anti tetanus antibody <0.01 IU/ml - Non protective, 0.01– 0.09 IU/ml - No reliable protection Anti pneumococcal anti body - Minimum accepted level 20 U/ml Binary outcomes were compared using chi-square test #Numeric outcomes were compared using Mann Witney U test ^a^DAS at baseline and at 24 weeks in rituximab group, *p* < 0.001 based on paired t-test ^b^DAS between baseline and at 24 weeks, in leflunomide group, *p* < 0.001 based on paired t-test

The EULAR responses were also high in both groups. At baseline, both groups had a high disease activity (DAS28 > 5.1) in 95% of the rituximab and 100% of the leflunomide group. At 24 weeks low disease activity or DAS < 3.2 as well as EULAR good response was seen in 40% of the rituximab and 42% of the leflunomide group respectively with none of the patients having DAS high disease activity (DAS >5.1) [Table 2]. The addition of either medication produced significant changes in disease activity scores from baseline level. None of the differences in clinical responses in any of the outcome criteria assessed in the two groups were statistically significant [Table 2].

**Fig. 2:**
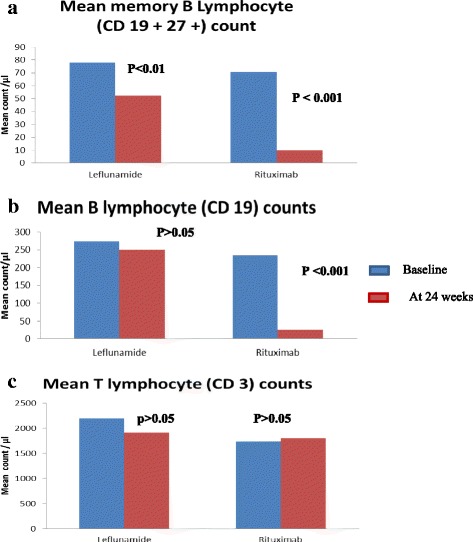
**a** **b** and **c** Measured B and T lymphocyte counts at baseline and at 24 weeks. **a** Memory B lymphocyte counts (CD 19+ 27+) at baseline and 24 weeks. Leflunomide shows significant difference in Memory B lymphocytes (CD19 + 27+) at 24 weeks compared to baseline (*P* < 0.01). Rituximab shows significant difference of Memory B lymphocytes (CD19 + 27+) at 24 weeks compared to baseline (*p* < 0.001). **b** Mean B lymphocyte counts (CD 19) at baseline and 24 weeks. Leflunomide shows no significant difference in B lymphocyte counts (CD19) at 24 weeks compared to baseline (*P* > 0.05). Rituximab shows significant difference in B lymphocyte counts (CD 19) at 24 weeks compared to baseline (*p* = <0.001). **c** T lymphocyte counts (CD 3) in patients at baseline and 24 weeks. Leflunomide shows no significant difference in T lymphocyte counts (CD 3) at 24 weeks compared to baseline (*P* > 0.05). Rituximab shows no significant difference in T lymphocyte counts (CD 3) at 24 weeks compared to baseline (*P* > 0.05). Data Analysed using non-parametric Wilcoxan Rank Sum test as paired samples

**Fig. 3:**
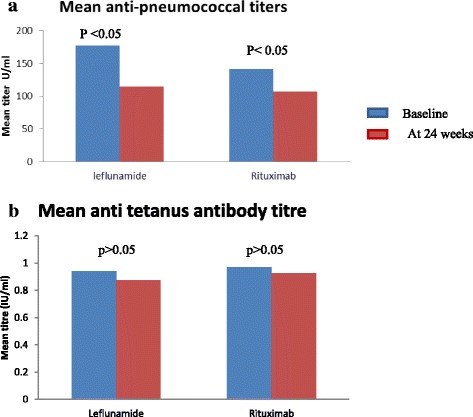
**a** and **b** Anti pneumococcal and anti tetanus antibody status at baseline and at 24 weeks. **a** Anti pneumococcal antibody titres at baseline and 24 weeks. Leflunomide shows significant difference of anti pneumococcal antibodies at 24 weeks compared to baseline (*P* < 0.05). Rituximab shows significant difference of anti pneumococcal antibodies at 24 weeks compared to baseline (*P* < 0.05) Anti pneumococcal anti body - Minimum accepted level - 20 U/ml. **b** Anti-tetanus antibody titres at baseline and 24 weeks. Leflunomide shows no significant difference of anti-tetanus antibodies at 24 weeks compared to baseline (*P* > 0.05). Rituximab shows no significant difference of anti tetanus antibodies at 24 weeks compared to baseline (*P* > 0.05). Data Analysed using non parametric Wilcoxan Rank Sum test as paired samples. Anti tetanus antibody <0.01 IU/ml Non protective, 0.01– 0.09 IU/ml No reliable protection

There were no significant differences in B cell, T cell or B cell memory percentages between the two groups at the start of the study. Compared to baseline, 24 week post-treatment levels showed the rituximab group having significant reduction in B cells (*p* < 0.001) and memory B cells (*p* < 0.001), [Fig. 2a and b] and pneumococcal antibody levels (*p* < 0.05) [Fig. 3b] with no significant change in T cells (*p* > 0.05) [Fig. 2c] or tetanus antibody levels (*p* > 0.05) [Fig. 3a]. The leflunomide group also showed significant change in memory B cells (*p* < 0.05) but T cells [Fig. 2] and tetanus antibody levels [Fig. 3b] did not show significant difference from baseline (*P* > 0.05). Both groups showed a significant reduction in pneumococcal antibody levels (*P* < 0.05) [Fig. 3a] and B memory cells (*P* < 0.01) [Fig. 2a]. There were no significant differences in other laboratory measurements in either group during the study period (rheumatoid factor, ESR, CRP, IgG, IgM levels and liver function tests).

There were five serious adverse events (SAE) in the rituximab group and three in the leflunomide group (including one death). In the rituximab group, four SAEs were due to infections, two patients developing lower respiratory tract infections, one having cellulitis in leg and the other having an infected toe, and all requiring IV antibiotics. In patients who had respiratory infections, the organism could not be identified but tuberculosis was excluded using the relevant investigations. In the leflunomide group, one patient developed an infective diarrhea (no organism isolated) and the other two patients had cardiac events. There were total of 3 cardiac events in both groups. In the rituximab group, one patient developed unstable angina at 16 weeks and was treated medically. In the leflunomide group, there were two cardiac events, one patient was admitted complaining of non-specific chest pain with elevated blood pressure and the other patient died of a myocardial infarction (MI). This patient with MI was admitted to hospital with a febrile illness and whilst in hospital developed an extensive anterior MI. The patient had multiple cardiovascular risk factors including long standing RA, prolonged NSAID use intermittently (more than 5 years), hypertension for 10 years and dyslipidemia. In the causality assessment the death was not considered to be due to study drug.

## Discussion

In this randomized double-blind controlled clinical trial among patients with rheumatoid arthritis refractory to conventional DMARDs, leflunomide-methotrexate combination was as efficacious as two infusions of low-dose rituximab (500 mg)-methotrexate combination, indicated by similar primary endpoints (ACR20) at 24 weeks. Although the steroids used in the protocol could have partly contributed to the responses seen in both groups, the previous studies using similar steroid regimens have shown that this is unlikely [16]. The response rates observed in this study are also higher than the rates reported from European trials using similar steroid regimens. The ACR 20, 50 and 70 achieved was 73%, 43% and 23% in the trial done by Edward et al. with 1000 mg rituximab [12] where similar steroid regimen was used. The study by Emery et al. using similar steroid regimen (100 mg methylprednisolone administered IV prior to rituximab infusions on days 1 and 15, and prednisone administered orally at 60 mg on days 2–7 and at 30 mg on days 8–14) has shown that glucocorticoid treatment had no significant effect on ACR20 response, through detailed statistical analysis on their data [16]. However they have shown that patients who received this regimen of glucocorticoids were more likely to achieve an ACR20 response early, (by week 4) than those who received no glucocorticoids. Therefore it is unlikely that the higher effects we observed were due to the use of high dose steroids in both arms.

The study by Emery et al. [16] used 2 doses of 500-mg infusions of rituximab with MTX in one arm and at week 24, ACR20, 50 and 70 was achieved in 55%, 33% and 13% respectively in that arm. The SERENE study [17] achieved 54.5% 26.3% and 9% rates of ACR 20, 50 and 70 with two doses of rituximab 500 mg. Thus achieving ACR 20, 50, 70 in 85%, 60% and 35% respectively in the low dose rituximab group in our study suggest a higher response in our population. Higher responses rates were also achieved for EULAR good response and DAS low activity in our study, (Table 2) compared to response rates reported in previous studies. In the SERENE study [17] the EULAR good response rates were seen only in 17.4% and 11.8% patients receiving two doses of 500 mg and 1 g of rituximab respectively. In the study by Emery et al. also, EULAR good response was achieved in only 28% and 14% patients receiving rituximab 500 mg and 1 g doses respectively [16]. Therefore our study showing DAS low activity and EULAR good response in 40% and 42% in the two arms are much higher compared to the response rates observed in other similar studies. This makes our results even more significant.

The trial comparing methotrexate-leflunomide therapy in non-responders to methotrexate [21] showed an ACR 20, 50,70 of 46%, 26% and 10% for the leflunomide-methotrexate group. Thus the responses seen in our methotrexate and leflunomide group of 85%, 64% and 32% for ACR 20, 50,70 responses observed in the leflunomide group are also comparatively higher in our population.

These data raise the question whether South Asian populations respond differently to these drug combinations than their European counterparts giving these higher response rates.

These results are also important as the response rate of low dose rituximab in our study is comparable to response rates seen with high dose standard rituximab therapy, reported in European trials [12, 13, 16, 17]. Some of the recent evidence from other studies also suggest that low dose rituximab (500 mg) has almost similar efficacy as the high dose of 1000 mg rituximab [17–19].

Rituximab causes B lymphopenia and a reduction in memory B-lymphocytes. Although rituximab produced B cell lymphopenia as expected, no measurable reduction from baseline levels were seen in tetanus antibody levels. However there was a significant reduction in pneumococcal antibody levels with both drugs [Fig. 3a]. The leflunomide group also showed a significant reduction in memory B cells [Fig. 2a] although B cells, T cells [Fig. 2b and c] and tetanus antibody levels [Fig. 2b] did not show a significant reduction from baseline. The observed changes in immune parameters with leflunomide are novel and need further characterization. Leflunomide showing comparable efficacy to rituximab could be explained to some extent by the significant reduction of memory B cells seen with leflunomide, similar to the effect produced by rituximab.

We observed a trend towards increased incidence of infections in the rituximab group with four patients requiring hospitalizations due to infections. Increased incidence of lower respiratory tract infections with rituximab has been reported from the European trials [15]. Although pneumococcal infections were not increased in our population, since pneumococcal antibody levels significantly reduced in both groups, possibility of reduced protection against pneumococcal infections with both treatments is a concern. However studies of rituximab, including those with long term follow up, in combination with other biologics and biologic registry data have shown that the incidence of serious infections with rituximab is low [25–28]. Our study also supports these findings, as there were no serious infections in our treatment population, which is reassuring as infections are more common in our setting.

No patients in our study developed tuberculosis during the study period. Like many countries in South and South-East Asia, TB is endemic in Sri Lanka and the use of a medication that does not increase the baseline risk of tuberculosis is advantageous.

Few patients from both groups had some cardiac events, which is well recognized among patients with RA. Significant increase in liver toxicity observed in some other reports [21] was not seen in the methotrexate-leflunomide group.

One of the limitations of our study is the small sample size, due to the limited availability of only 40 vials of rituximab for this study. Though the trial was small, it had 70% power to detect a 30% difference in the treatment groups. It is possible that with a larger sample size, smaller differences between the two groups could have been detected. Further observations from larger clinical trials may help to confirm the findings from this study.

This study suggests that comparatively cheaper therapies such as methotrexate-leflunomide combination or low dose rituximab-methotrexate combination may be beneficial in South Asian patients who are not responding to standard first line treatment in RA.

The trial data has significant beneficial cost implications for treatment of patients with refractory rheumatoid arthritis, especially in resource limited settings in South Asian regions. The cost of two infusions of 1000 mg rituximab was Sri Lankan Rupees (SLRs) 744,000(US$ 5700), according to the cost of rituximab when this trial was done. Annual costs for TNFi are also similar to this figure or even more expensive. Two infusions of rituximab of 500 mg halves this cost, to SLRs 372,000(US $ 2850). Leflunomide at a dose of 20 mg per day drastically reduces this cost to only SLRs 25,200 (US $ 192) for one year of treatment. Thus our finding that the response rates were similar to those reported in clinical trials using high-dose rituximab or the TNFi in European patients has highly favorable cost implications for patients living in a developing country. We highlight that with the lower dose of rituximab used, the biologic, which is relatively affordable in South East Asia is low dose rituximab. As this trial shows that Leflunomide is as efficacious as low dose rituximab given in combination with methotrexate and since recent studies [22] have shown the safety of Leflunomide-methotrexate combination, this combination could be recommended prior to trying the biologics in patients with refractory RA in South East Asia.

## Conclusion

This study showed that both leflunomide and low dose rituximab were equally efficacious in controlling disease activity when added to methotrexate in Sri Lankan patients with refractory RA. This has significant cost implications as leflunomide-methotrexate combination is much cheaper compared to low-dose rituximab-methotrexate combination. The lower costs of both these two treatment options should enable more patients with refractory rheumatoid arthritis to be treated successfully in resource-limited settings.

## Abbreviations

ACR: American college of rheumatology
CTLA4: Cytotoxic T lymphocyte Antigen-4
DAS: Disease activity score
DMARDs: Disease modifying anti rheumatic drugs
ELISA: Enzyme linked immunosorbent assay
ESR: Erythrocyte sedimentation rate
EULAR: European league against rheumatism
HAQ: Health-assessment questionnaire
MI: Myocardial infarction
NHSL: National Hospital of Sri Lanka
NSAIDS: Non-steroidal anti-inflammatory drugs
RA: rheumatoid arthritis
SAE: Serious adverse events
SERENE: Study Evaluating Rituximab's Efficacy in MTX iNadequate rEsponders
SLCTR: Sri Lanka clinical trials registry
SLRs: Sri Lankan Rupees
SMILE: Safety of Methotrexate In combination with LEflunomide in rheumatoid arthritis
TB: Tuberculosis
TNFi: Tumor necrosis factor inhibitors
US$: United States Dollars

## References

[1] Saag KG, Teng GG, Patkar NM, Anuntiyo J, Finney C, Curtis JR (2008). American College of Rheumatology 2008 recommendations for the use of nonbiologic and biologic disease-modifying antirheumatic drugs in rheumatoid arthritis. Arthritis Rheum.

[2] Singh JA, Furst DE, Bharat A, Curtis JR, Kavanaugh AF, Kremer JM (2012). 2012 update of the 2008 American College of Rheumatology recommendations for the use of disease-modifying antirheumatic drugs and biologic agents in the treatment of rheumatoid arthritis. Arthritis Care Res.

[3] Administration USFDA Drug Safety Communication: new boxed warning for severe liver injury with arthritis drug Arava (leflunomide). 2010. www.fda.gov/Drugs/DrugSafety/ucm218679.html (accessed 15 May 2015).

[4] van Vollenhoven RF, Ernestam S, Geborek P, Petersson IF, Coster L, Waltbrand E (2009). Addition of infliximab compared with addition of sulfasalazine and hydroxychloroquine to methotrexate in patients with early rheumatoid arthritis (Swefot trial): 1-year results of a randomised trial. Lancet.

[5] St. Clair EW, van der Heijde DMFM, Smolen JS, Maini RN, Bathon JM, Emery P (2004). Combination of infliximab and methotrexate therapy for early rheumatoid arthritis: a randomized, controlled trial. Arthritis Rheum.

[6] Seong S-S, Choi C-B, Woo J-H, Bae KW, Joung C-L, Uhm W-S (2007). Incidence of tuberculosis in Korean patients with rheumatoid arthritis (RA): effects of RA itself and of tumor necrosis factor blockers. J Rheumatol.

[7] Wallis RS, Broder M, Wong J, Lee A, Hoq L (2005). Reactivation of latent granulomatous infections by infliximab. Clin Infect Dis.

[8] Keane J (2005). TNF-blocking agents and tuberculosis: new drugs illuminate an old topic. Rheumatology.

[9] Tubach F, Salmon D, Ravaud P, Allanore Y, Goupille P, Bréban M (2009). Risk of tuberculosis is higher with anti–tumor necrosis factor monoclonal antibody therapy than with soluble tumor necrosis factor receptor therapy: the three-year prospective french research axed on tolerance of biotherapies registry. Arthritis Rheum.

[10] McGonagle D, Tan AL, Madden J, Taylor L, Emery P (2008). Rituximab use in everyday clinical practice as a first-line biologic therapy for the treatment of DMARD-resistant rheumatoid arthritis. Rheumatology (Oxford).

[11] Buch MH, Smolen JS, Betteridge N, Burmester G, Darner T, Ferraccioli G (2011). Updated consensus statement on the use of rituximab in patients with rheumatoid arthritis. Ann Rheum Dis.

[12] Edwards JC, Szczepanski L, Szechinski J, Filipowicz-Sosnowska A, Emery P, Close DR (2004). Efficacy of B-cell-targeted therapy with rituximab in patients with rheumatoid arthritis. N Engl J Med.

[13] Cohen SB, Emery P, Greenwald MW, Dougados M, Furie RA, Genovese MC (2006). Rituximab for rheumatoid arthritis refractory to anti-tumor necrosis factor therapy: results of a multicenter, randomized, double-blind, placebo-controlled, phase III trial evaluating primary efficacy and safety at twenty-four weeks. Arthritis Rheum.

[14] van Vollenhoven RF, Emery P, Bingham CO, Keystone EC, Fleischmann RM, Furst DE, et al. Long-term safety of rituximab in rheumatoid arthritis: 9.5-year follow-up of the global clinical trial programme with a focus on adverse events of interest in RA patients. Ann Rheum Dis. 2012; doi:10.1136/annrheumdis-2012-201956. 10.1136/annrheumdis-2012-201956 PMC3756452 23136242

[15] Rubbert-Roth A, Tak PP, Zerbini C, Tremblay J-L, Carreño L, Armstrong G (2010). Efficacy and safety of various repeat treatment dosing regimens of rituximab in patients with active rheumatoid arthritis: results of a phase III randomized study (MIRROR). Rheumatology.

[16] Emery P, Fleischmann R, Filipowicz-Sosnowska A, Schechtman J, Szczepanski L, Kavanaugh A (2006). The efficacy and safety of rituximab in patients with active rheumatoid arthritis despite methotrexate treatment: results of a phase IIB randomized, double-blind, placebo-controlled, dose-ranging trial. Arthritis Rheum.

[17] Emery P, Deodhar A, Rigby WF, Isaacs JD, Combe B, Racewicz AJ, Latinis K, Abud-Mendoza C, Szczepański LJ, Roschmann RA, Chen A (2010). Efficacy and safety of different doses and retreatment of rituximab: a randomised, placebo-controlled trial in patients who are biological naive with active rheumatoid arthritis and an inadequate response to methotrexate (study evaluating Rituximab's efficacy in MTX iNadequate rEsponders (SERENE)). Ann Rheum Dis..

[18] Bredemeier M, Oliveira FK, Rocha CM (2014). Low-versus high-dose Rituximab for rheumatoid arthritis: a systematic review and meta-analysis. Arthritis Care Res.

[19] Chatzidionysiou K, Lie E, Nasonov E, Lukina G, Hetland ML, Tarp U, Ancuta I, Pavelka K, Nordström DC, Gabay C, Canhão H (2016). Effectiveness of two different doses of rituximab for the treatment of rheumatoid arthritis in an international cohort: data from the CERERRA collaboration. Arthritis Res Ther.

[20] Kremer JM, Genovese MC, Cannon GW, Caldwell JR, Cush JJ, Furst DE (2002). Concomitant leflunomide therapy in patients with active rheumatoid arthritis despite stable doses of methotrexate. A randomized, double-blind, placebo-controlled trial. Ann Intern Med.

[21] van Roon EN, Tim LTA, Houtman NM, Spoelstra P, Brouwers JR (2004). Leflunomide for the treatment of rheumatoid arthritis in clinical practice. Drug Saf.

[22] Bird P, Griffiths H, Tymms K, Nicholls D, Roberts L, Arnold M, Burnet S, de Jager J, Scott J, Zochling J, Littlejohn G (2013). The SMILE study—safety of methotrexate in combination with leflunomide in rheumatoid arthritis. J Rheumatol.

[23] Arnett FC, Edworthy SM, Bloch DA, Mcshane DJ, Fries JF, Cooper NS, Healey LA, Kaplan SR, Liang MH, Luthra HS, Medsger TA (1988). The American rheumatism association 1987 revised criteria for the classification of rheumatoid arthritis. Arthritis Rheum.

[24] Rheumatoid arthritis: No advantage of leflunomide loading dose. Nat Rev Rheumatol. 2013;9(4):196.

[25] van Vollenhoven RF, Emery P, Bingham CO, 3rd, Keystone EC, Fleischmann RM, Furst DE, et al. Long-term safety of rituximab in rheumatoid arthritis: 9.5-year follow-up of the global clinical trial programme with a focus on adverse events of interest in RA patients. Ann Rheum Dis. 2012;72:1–7. 10.1136/annrheumdis-2012-201956 PMC3756452 23136242

[26] Keystone EC, Cohen SB, Emery P, Kremer JM, Dougados M, Loveless JE (2012). Multiple courses of rituximab produce sustained clinical and radiographic efficacy and safety in patients with rheumatoid arthritis and an inadequate response to 1 or more tumor necrosis factor inhibitors: 5-year data from the REFLEX study. J Rheumatol.

[27] Mekinian A, Ravaud P, Hatron P, Larroche C, Leone J, Gombert B (2012). Efficacy of rituximab in primary Sjögren's syndrome with peripheral nervous system involvement: results from the AIR registry. Ann Rheum Dis.

[28] Rigby WFC, Mease PJ, Olech E, Ashby M, Tole S (2013). Safety of rituximab in combination with other biologic disease-modifying antirheumatic drugs in rheumatoid arthritis: an open-label study. J Rheumatol.

